# Advancements in differentiation of induced pluripotent stem cells into specialized neuronal subtypes

**DOI:** 10.4103/NRR.NRR-D-25-00630

**Published:** 2025-10-30

**Authors:** Selena Setsu, Hideyuki Okano, Satoru Morimoto

**Affiliations:** 1 Laboratory of RNA Function, Institute for Quantitative Biosciences, The University of Tokyo, Tokyo, Japan; 2 Department of Computational Biology and Medical Sciences, Graduate School of Frontier Sciences, The University of Tokyo, Tokyo, Japan; 3 Keio University Regenerative Medicine Research Center, Kanagawa, Japan

**Keywords:** cell therapy, disease modelling, dopaminergic neurons, drug screening, glutamatergic neurons, induced pluripotent stem cells, motor neurons, neuronal differentiation, Purkinje cells, regenerative medicine, sensory neurons

## Abstract

The ability to generate specialized human neurons from induced pluripotent stem cells has revolutionized neuroscience, regenerative medicine, and drug discovery. Since their discovery, induced pluripotent stem cells have emerged as an ethically favorable and versatile platform to model human neurological diseases, offering new insights beyond traditional animal models. In the past decade, rapid advances have enabled the efficient differentiation of induced pluripotent stem cells into diverse neuronal subtypes, including glutamatergic neurons, GABAergic neurons, dopaminergic neurons, serotonergic neurons, motor neurons, sensory neurons, Purkinje cells, sympathetic neurons, parasympathetic neurons, and noradrenergic neurons. Tailored combinations of developmental signaling molecules, transcription factor programming, and small molecule modulation have dramatically improved the reproducibility, scalability, and functional maturity of these differentiated neurons. These advancements are particularly timely as they underpin the next generation of disease modelling platforms, high-throughput drug screening systems, and emerging cell-based therapies for conditions such as Parkinson’s disease, amyotrophic lateral sclerosis, epilepsy, and Alzheimer’s disease. Moreover, the field is moving toward standardized, chemically defined protocols and improved validation pipelines, including electrophysiological assays and molecular profiling, to ensure the authenticity and maturity of induced pluripotent stem cell-derived neurons. Notably, recent breakthroughs in sympathetic and parasympathetic neuron derivation are expanding the scope of induced pluripotent stem cell technology into autonomic nervous system research and cardiac neuromodulation studies. However, challenges remain, including variability across induced pluripotent stem cell lines, incomplete neuronal maturation, and scalability constraints for clinical-grade applications. Addressing these hurdles through optimization of patterning cues, co-culture systems, and advanced bioprocessing strategies will be crucial to realizing the full translational potential of induced pluripotent stem cell-derived neurons. Collectively, the methodologies and developments summarized here mark a major step toward achieving faithful, efficient, and scalable generation of human neurons *in vitro*, laying the foundation for personalized neurology and regenerative medicine.

## Introduction

The development of induced pluripotent stem cells (iPSCs) is rooted in decades of reprogramming research, beginning with John Gurdon’s landmark 1962 nuclear transfer experiments, which demonstrated that differentiated cells retain the full genetic potential to develop into an organism. This foundational concept paved the way for the groundbreaking work by Yamanaka and Takahashi in 2006, who identified four transcription factors—OCT4, SOX2, KLF4, and c-MYC—that could reprogram somatic cells, such as dermal fibroblasts, into pluripotent stem cells functionally equivalent to embryonic stem cells (Takahashi and Yamanaka, 2006; Takahashi et al., 2007). This discovery revolutionized regenerative medicine, offering an ethically acceptable alternative to human embryonic stem cells and earning the 2012 Nobel Prize. Subsequent advances have focused on improving the safety and efficiency of iPSC generation through non-integrative techniques (e.g., mRNA transfection and Sendai virus) and, more recently, complete chemical reprogramming—achieved first in mice and now in humans—as a safer, non-genetic alternative (Hou et al., 2013; Kogut et al., 2018; Guan et al., 2022; Kunitomi et al., 2022). iPSCs now serve as a powerful platform for disease modeling, drug screening, tissue regeneration, and aging reversal. In particular, the directed differentiation of iPSCs into defined neuronal subtypes has become indispensable for studying neurodevelopment, disease pathogenesis, and patient-specific genetic variants, and the field continues to progress rapidly toward clinical translation as we approach the 20th anniversary of their discovery (Fujimori et al., 2018; Kim et al., 2022; Okano and Morimoto, 2022; Okano et al., 2023).

The ability to produce human neurons *in vitro* also addresses key limitations of conventional animal models, which often fail to recapitulate the cellular complexity and genetic background of human neurological disorders. iPSC-derived neurons permit direct evaluation of disease-causing mutations, environmental influences, and pharmacological responses in a human context. They further underpin emerging precision-medicine strategies, whereby patient-specific neurons are used to predict individual therapeutic responses and guide tailored treatment regimens.

The human nervous system comprises multiple neuronal classes, each with specialized functions in the central and peripheral nervous systems. Major categories include glutamatergic, GABAergic, dopaminergic, serotonergic, noradrenergic neurons, motor, sensory, sympathetic, parasympathetic neurons, and cerebellar Purkinje cells. Dysfunction within any of these populations can precipitate disorders such as Parkinson’s disease, Alzheimer’s disease, Huntingtin’s disease, epilepsy, motor neuron disease and many other neurological disorders including neuropsychiatric disorders. Robust, reproducible protocols for generating each subtype from iPSCs are therefore essential for both fundamental neuroscience and translational applications, including disease modelling, high-throughput drug screening, and cell-based therapies. Importantly, the prospect of cell-based therapies for neurological disorders is becoming increasingly realistic, with multiple clinical trials currently underway (Svendsen and Svendsen, 2024; Kirkeby et al., 2025). Among these, cell therapy for Parkinson’s disease is at the forefront of clinical translation.

While iPSCs represent a powerful resource, it is important not to overlook the ethical dimensions of iPSC-based research. iPSCs, initially hailed as an ethical alternative to embryonic stem cells, circumvent the need for embryo destruction. However, they introduce new ethical complexities, including concerns over informed consent, donor privacy, long-term storage and use, and the risks posed by genetic instability and tumorigenicity. Emerging applications, such as gamete derivation and embryo-like structures, raise further moral considerations. Additionally, issues surrounding commercialization and global disparities in access require ongoing attention. As iPSC technologies progress toward clinical implementation, robust governance frameworks, equitable access policies, anonymization of personal information, and transparent consent processes will be essential to ensure responsible and socially aligned innovation.

This review surveys progress over the past decade in driving iPSC differentiation toward specialized neuronal lineages. We compare current methodologies, highlight recurring technical challenges, and discuss their utility in disease modelling and regenerative medicine. By consolidating recent advances, we aim to provide a comprehensive resource that will inform future work and accelerate the translation of iPSC technology into neuroscience research and clinical practice.

## Search Strategy

Studies included in this narrative review, published primarily between 2015 and 2025, were identified through PubMed searches using relevant keywords. Search terms comprised iPSC, neural induction, glutamatergic neurons, GABAergic neurons, Purkinje cells, cholinergic neurons, motor neurons, dopaminergic neurons, serotonergic neurons, sensory neurons, norepinephrine neurons, noradrenergic neurons, sympathetic neurons, and parasympathetic neurons. In addition, seminal older studies are cited to acknowledge and reference groundbreaking work that laid the foundation for current advancements.

## Scope of This Review

This article surveys the state-of-the-art two-dimensional differentiation strategies that steer human iPSCs toward nine clinically relevant neuronal lineages: glutamatergic projection neurons, GABAergic interneurons, cerebellar Purkinje cells, spinal motor neurons, locus coeruleus noradrenergic neurons, peripheral sensory neurons, midbrain dopaminergic neurons, raphe serotonergic neurons, and sympathetic and parasympathetic autonomic neurons. Although these groups do not capture the full diversity of the nervous system, they encompass major classes with great basic and translational significance. For each lineage-specific section, we address three themes: (1) Developmental rationale – a concise overview of the embryological signaling cascades that specify the target phenotype *in vivo*. (2) Key protocols – A synthesis of established and emerging two-dimensional methods, highlighting indispensable transcription factors, morphogens, small molecules, and culture parameters. (3) Applications and challenges – current uses in disease modeling, drug discovery, and prospective cell therapies, along with persistent bottlenecks and future directions.

By consolidating these methodologies, we aim to equip both newcomers and experienced investigators with a practical reference for designing experiments that generate defined neuronal subtypes from human iPSCs.

Here, we briefly note that variability and heterogeneity remain major limitations in iPSC differentiation. Factors such as donor genetic background, somatic mutations, epigenetic memory, and culture conditions lead to inconsistent outcomes in efficiency, identity, and maturation, even with standardized protocols. These issues hinder reproducibility and complicate disease modeling and therapeutic development. Heterogeneous cultures containing off-target or immature cells can also obscure biological signals. As this review does not focus on variability, we refer readers to a detailed discussion provided elsewhere for in-depth analysis of causes, consequences, and mitigation strategies (Volpato and Webber, 2020).

## General Strategies for Differentiating Induced Pluripotent Stem Cells into Neurons

### Neural induction

Neural induction converts pluripotent iPSCs into expandable neural stem/progenitor cells. Classically, the formation of neural ectoderm *in vivo* depends on bone morphogenetic protein (BMP) signaling inhibition. Mimicking this process *in vitro*, many protocols apply dual-SMAD inhibition—a technique introduced by Chambers et al. (2009) that combines Noggin, LDN-193189 (LDN), DMH1 or dorsomorphin (BMP-pathway inhibitors) and SB431542 (SB, transforming growth factor-β-pathway inhibitor) to drive the neural lineage (Chambers et al., 2009).

This stage generates neural stem/progenitor cells that can be passaged repeatedly while retaining neurogenic and gliogenic potential. Various adherent or three-dimensional (e.g., embryoid-body or organoid) formats exist, each with advantages and limitations for purity, patterning, and scalability.

### Patterning, regional specification, and transcription-factor programming

To move beyond generic neural identities, researchers leverage additional cues that pattern cells along the anteroposterior and dorsoventral axes. For instance: (1) WNT signaling – promotes posterior fates (spinal cord, hindbrain); (2) Retinoic acid – cooperates with WNT to generate spinal/hindbrain derivatives such as motor neurons and some sensory interneurons (Okada et al., 2004; Janesick et al., 2015); (3) Fibroblast growth factors (FGFs) – context-dependent; can maintain neural progenitor cells (NPCs) or bias them anteriorly/posteriorly according to dose and timing; (4) Sonic hedgehog (SHH) – ventralizing morphogen that specifies ventral spinal-cord neural progenitors (motor neurons), and mid-brain dopaminergic neurons.

**Figure 1:**
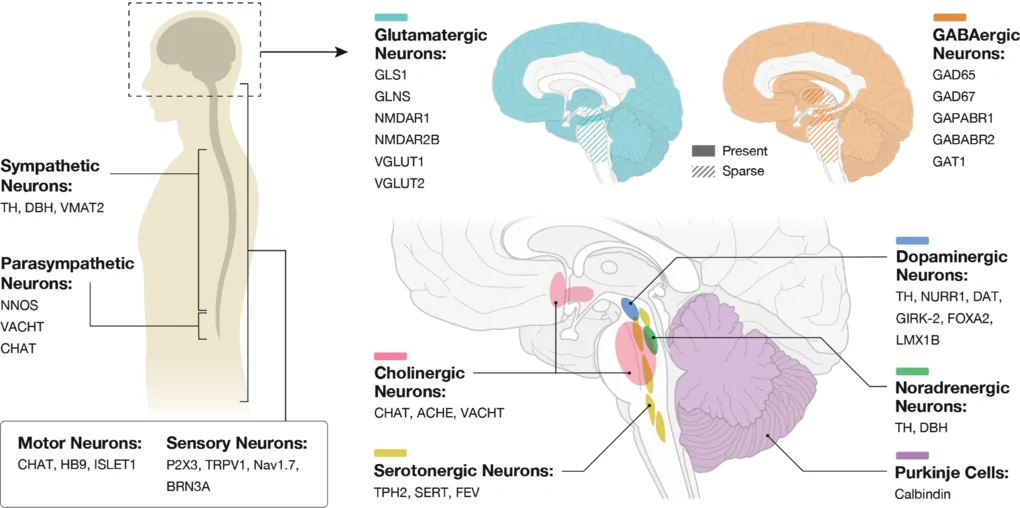
Focused neuronal subtypes in this study and their representative markers. This schematic illustrates key neuronal subtypes that can be generated from human iPSCs, mapped according to their anatomical origins within the central and peripheral nervous systems. Central nervous system lineages include forebrain (glutamatergic, GABAergic), midbrain (dopaminergic), hindbrain (serotonergic, noradrenergic), spinal cord (motor neurons), and cerebellum (Purkinje cells). Peripheral nervous system derivatives include sensory, sympathetic, and parasympathetic neurons. Cholinergic neurons are found in both domains. This figure emphasizes the diversity of neuronal fates accessible through iPSC differentiation and their relevance to modeling region-specific functions and disorders across the nervous system. Illustration created by LAIMAN Inc. and used with permission under purchased license. AChE: Acetylcholinesterase; BRN3A: brain-specific homeobox/POU domain protein 3A; ChAT: choline acetyltransferase; DAT: dopamine transporter; DBH: dopamine beta-hydroxylase; FEV: fifth Ewing variant (also known as PET1, an ETS domain transcription factor); FOXA2: forkhead box A2; GABABR1: gamma-aminobutyric acid type B receptor subunit 1; GABABR2: gamma-aminobutyric acid type B receptor subunit 2; GAD65: glutamate decarboxylase 65; GAD67: glutamate decarboxylase 67; GAT1: GABA transporter 1; GIRK-2: G protein-coupled inwardly-rectifying potassium channel 2; GLNS: glutaminase; HB9: homeo box HB9 (MNX1, motor neuron and pancreas homeobox 1); ISLET1: ISL LIM homeobox 1; LMX1B: LIM homeobox transcription factor 1 beta; Nav1.7: voltage-gated sodium channel subunit alpha Nav1.7 (SCN9A, sodium voltage-gated channel alpha subunit 9); NMDAR1: N-methyl-D-aspartate receptor subunit 1 (GRIN1, glutamate ionotropic receptor NMDA type subunit 1); NMDAR2B: N-methyl-D-aspartate receptor subunit 2B (GRIN2B1, glutamate ionotropic receptor NMDA type subunit 2B); NNOS: neuronal nitric oxide synthase 1; NURR1: nuclear receptor related 1; P2X3: P2X purinoceptor 3; SERT: serotonin transporter; TH: tyrosine hydroxylase; TPH2: tryptophan hydroxylase 2; TRPV1: transient receptor potential vanilloid 1; VAChT: vesicular acetylcholine transporter; VGLUT1: vesicular glutamate transporter 1 (SLC17A7, solute carrier family 17 member 7); VGLUT2: vesicular glutamate transporter 2 (SLC17A6, solute carrier family 17 member 6); VMAT2: vesicular monoamine transporter 2 (SLC18A2, solute carrier family 18 member A2).

In essence, each neuronal subtype is specified by coupling neural induction with precisely timed, dose-controlled regional cues that recapitulate the *in vivo* milieu (**Figure 1** and **Additional Figure 1**).

Researchers have sought to improve efficiency and specificity by using transcription factors to directly guide iPSCs into defined subtypes. For example, a neuronal basic helix-loop-helix factor, neurogenin2 (NGN2), has been widely used to produce excitatory neurons (Zhang et al., 2013), including cortical-like neurons (Hulme et al., 2022). Besides NGN2, tailored combinations of lineage-defining factors can rapidly yield other phenotypes, reducing reliance on extracellular cues and enabling robust, reproducible protocols (**Additional Figure 2**).

Various methods have been developed to overexpress transcription factors for neuronal differentiation, each with specific advantages and limitations. The most reproducible and tightly controlled approach involves establishing doxycycline-inducible cell lines through targeted gene editing, typically into safe harbor loci such as AAVS1. This method allows precise regulation of transcription factor expression and ensures consistency across experiments. However, generating such inducible lines is time-consuming and resource-intensive, making it impractical for studies involving multiple iPSC lines. Alternatively, systems like the piggyBac transposon and lentiviral vectors allow stable integration and long-term expression of transcription factors. While these approaches are relatively efficient, they carry the risk of random genomic integration, which may interfere with endogenous gene function. Non-integrating strategies such as Sendai virus offer a safer alternative. As an RNA virus, Sendai virus does not integrate into the host genome and provides high transfection efficiency with minimal genomic disruption. Despite these advantages, the technical complexity of producing and handling Sendai virus remains a limiting factor. Plasmid transfection offers a simple, non-integrating option for transient expression but generally results in lower efficiency and less consistent outcomes. Ultimately, the choice of method depends on the specific experimental context, balancing reproducibility, safety, efficiency, and scalability.

### Maturation and functional validation

Neuronal differentiation from iPSCs requires not only subtype-specific markers but also functional maturity, which is critical for disease modelling and cell-therapy applications. Maturation can be promoted by: (1) Extended culture: Weeks–months are often needed for full electrophysiological and synaptic competence; (2) co-culture with astrocytes or complementary neurons: glial support accelerates maturation; (3) neurotrophic factors: brain-derived neurotrophic factor (BDNF), glial cell line-derived neurotrophic factor (GDNF), neurotrophin-3 (NT-3) and ciliary neurotrophic factor (CNTF) enhance survival and synaptogenesis

Validation typically involves: (1) Immunocytochemistry: Detection of pan-neuronal markers such as βIII-tubulin and microtubule-associated protein 2 (MAP2), along with subtype-specific markers (**Additional Figure 2**), to confirm neuronal identity and differentiation state. (2) Gene expression analysis: reverse transcription quantitative polymerase chain reaction or RNA sequencing is used to evaluate lineage-specific transcriptional profiles and verify the expression of genes appropriate for the intended neuronal subtype. (3) Electrophysiological characterization: Patch-clamp recordings are conducted to assess intrinsic membrane properties, action potential generation, and synaptic activity. Alternatively, multielectrode array platforms can be used to monitor extracellular electrical activity across neuronal networks. (4) Calcium imaging: Measurement of intracellular calcium transients allows monitoring of neuronal excitability, spontaneous activity, and network-level dynamics.

## Glutamatergic Neurons

Glutamatergic neurons, the principal excitatory projection cells of the mammalian cortex, make up the majority of cortical projection neurons in the mammalian forebrain. Embryonically, they originate from radial-glial progenitors in the dorsal telencephalic ventricular zone. The transcriptional and signaling landscape that guides glutamatergic lineage commitment includes key transcription factors such as PAX6, EMX2, and NGN2.

### Protocols for differentiating induced pluripotent stem cells into glutamatergic neurons

Early protocols often relied on spontaneous embryoid-body differentiation, producing heterogeneous neural populations. More refined methods now incorporate stage-specific inhibition of BMP and transforming growth factor-β pathways (dual-SMAD inhibition) to drive efficient neural induction, followed by dorsal-forebrain patterning (Chambers et al., 2009; Shi et al., 2012; Cao et al., 2017b).

A typical workflow comprises: (1) Neural induction: Dual-SMAD inhibition with Noggin, LDN, DMH1, or dorsomorphin plus SB in chemically defined medium. (2) Neural-progenitor expansion: NPCs are expanded with FGF2 to maintain proliferative capacity and dorsal-telencephalic identity. (3) Dorsal-forebrain specification: Avoidance of retinoic acid and/or use of WNT inhibitors (e.g., XAV-939) supports anterior cortical fate. (4) Terminal differentiation: NPCs are plated with BDNF, GDNF, NT-3, and cyclic adenosine monophosphate (cAMP) analogues (e.g., dbcAMP) to promote neurite outgrowth and synaptogenesis.

Glutamatergic identity is confirmed by immunostaining for vGLUT1, typically associated with cortical and hippocampal neurons, or vGLUT2, commonly expressed in thalamic and brainstem neurons, depending on the intended regional specification, and by electrophysiological detection of excitatory postsynaptic currents. Application of aminomethylphosphonic acid- or N-methyl-D-aspartate-receptor agonists elicits depolarizing currents, indicative of functional glutamatergic transmission.

Derived neurons express cortical-layer markers such as TBR1 (layer VI), CTIP2 (layer V), and SATB2 (layers II–IV). Cyclopamine-mediated SHH inhibition further enriches cortical glutamatergic identity while suppressing ventral GABAergic fates (Cao et al., 2017b).

The most widely used rapid approach is forced NGN2 expression (Zhang et al., 2013). NGN2 over-expression accelerates differentiation, yielding morphologically and electro physiologically mature-like neurons within 2–3 weeks (Ho et al., 2016; Wang et al., 2017; Fernandopulle et al., 2018; Nehme et al., 2018; Ishikawa et al., 2020; Ang et al., 2024; Shan et al., 2024). Although NGN2 overexpression is a simple and efficient method for generating functional neurons, it is known to produce a heterogeneous population that includes other neural subtypes, such as cholinergic neurons marked by ISL1 expression. More recent studies have aimed to enhance glutamatergic purity by co-expressing additional region-specific transcription factors. Notably, overexpression of EMX1 has been shown to significantly increase the proportion of glutamatergic neurons, yielding up to 95% vGLUT1-positive cells among MAP2-positive neurons, while simultaneously suppressing cholinergic markers such as ISL1, vesicular acetylcholine transporter (VAChT), and choline acetyltransferase (ChAT) (Ang et al., 2024).

### Challenges and applications in disease modelling

iPSC-derived glutamatergic neurons have been pivotal for modelling neurodevelopmental and neuropsychiatric disorders, including autism spectrum disorder, Alzheimer’s disease, and schizophrenia (Marchetto et al., 2010; Brennand et al., 2011; Israel et al., 2012; Habela et al., 2016; Yamamoto et al., 2021; Kondo et al., 2022; Zhang et al., 2022a; Perrottelli et al., 2024; Triebelhorn et al., 2024). They allow interrogation of disease-specific phenotypes such as dendritic-spine abnormalities, excitatory/inhibitory imbalance, and synaptic-plasticity defects.

Key challenges include: (1) Achieving reproducible layer-specific identities and recapitulating *in vivo* cortical lamination; (2) Balancing physiological complexity with culture homogeneity – organoids provide three-dimensional architecture but increase variability; (3) Promoting long-term functional maturation.

Co-culture of glutamatergic and GABAergic neurons is increasingly employed to foster balanced network activity (Xu et al., 2016a; Mossink et al., 2022; Wang et al., 2022, 2023). Balancing *in vivo*-like complexity with experimental reproducibility remains a central objective for the field.

## GABAergic Neurons

In the mammalian forebrain, most inhibitory interneurons that employ γ-aminobutyric acid (GABA) as their neurotransmitter arise from the medial, lateral, or caudal ganglionic eminence (MGE, LGE, CGE) in the ventral telencephalon. Key transcription factors guiding interneuron fate include NKX2.1 for MGE-derived interneurons—which give rise to parvalbumin- and somatostatin-expressing subtypes—and GSX2 for LGE lineages. Graded SHH signaling plays a major role in specifying ventral identities, in contrast to the dorsal‐forebrain specification required for glutamatergic neurons.

### Protocols for differentiating induced pluripotent stem cells into GABAergic neurons

Because default neuralization favors dorsal fates, generating GABAergic interneurons requires ventralizing cues (Liu et al., 2013; Maroof et al., 2013; Nicholas et al., 2013; Shao et al., 2019; Pilotto et al., 2023, 2024): (1) Neural induction: dual-SMAD inhibition to obtain forebrain-like NPCs. (2) Ventral patterning: addition of SHH or small-molecule agonists (SAG [smoothened agonist], purmorphamine) and, where required, FGF8 or FGF2 to promote MGE- or CGE-like identity. NKX2.1 expression marks successful MGE specification. (3) Progenitor expansion: culture in EGF/FGF2 or insulin-like growth factor 1 (IGF-1) to maintain proliferation. (4) Maturation: withdrawal of mitogens and supplementation with BDNF, GDNF, and other trophic factors. Over several weeks, cells up-regulate GAD67 and subtype markers such as parvalbumin or somatostatin.

Resultant interneurons are identified by GABA, GAD65/67 and—where appropriate—NKX2.1 (progenitors) plus PV or SST (mature subtypes), using immunocytochemistry or RNA sequencing. Electrophysiologically, these interneurons exhibit inhibitory postsynaptic currents, which are commonly assessed in co-culture with excitatory neurons (Xu et al., 2016a, Mossink et al., 2022, Wang et al., 2022, 2023). Recent protocols have achieved high efficiency in generating GABAergic neurons, with over 80% of cells expressing GABA or GAD65/67, as confirmed by both molecular markers and functional assays (**Additional Figures 1** and **2**, GABAergic neuron). Earlier patterning-based methods, while effective, often required extended differentiation periods, typically exceeding 60–80 days, and yielded heterogeneous populations of interneuron subtypes. In contrast, Pilotto et al. (2024) recently introduced an improved patterning-based protocol that significantly shortens the differentiation timeline to just 28 days while maintaining high efficiency, with 80% of MAP2-positive cells expressing GABA. In parallel, transcription factor-based programming using factors such as ASCL1, DLX2, and LHX6 has also proven successful in accelerating differentiation and improving subtype specificity (Colasante et al., 2015; Sun et al., 2016; Yang et al., 2017; Barretto et al., 2020). Together, these advances offer complementary strategies to generate GABAergic neurons more rapidly and with greater precision.

### Challenges and applications in disease modelling

Dysfunction of GABAergic interneurons is implicated in Huntington’s disease, schizophrenia, epilepsy, and autism (Russo et al., 2019; Schuster et al., 2019, 2024; Hirose et al., 2020; McNeill et al., 2020; Scalise et al., 2022; Wu et al., 2024c).

Among GABAergic classes, medium spiny neurons of the striatum are of particular interest for Huntington’s disease models because medium spiny neurons undergo selective degeneration (Nekrasov et al., 2016; Hunt et al., 2017; Victor et al., 2018; Grigor’eva et al., 2020). iPSC-derived interneurons also hold promise as cell therapies for refractory epilepsy and neurodevelopmental disorders characterized by diminished inhibitory tone (Zhu et al., 2018, 2023; Gonzalez-Ramos et al., 2021; Zhang et al., 2022b; Bershteyn et al., 2023). Notably, in 2024, a clinical study was initiated to evaluate the safety and efficacy of transplanting iPSC-derived inhibitory interneurons into patients with epilepsy (Neurona Therapeutics, 2024), marking a key step toward therapeutic application of these cells.

## Purkinje Cells

Purkinje cells are large GABAergic neurons located in the cerebellar cortex, forming a crucial node in the motor-coordination circuit. During embryogenesis they arise from ventricular-zone progenitors at the dorsal hindbrain (rhombic-lip) interface. Early specification of Purkinje progenitors involves transcription factors such as PTF1A, while their unique morphology, extensive dendritic arborization, and synaptic connectivity mature through interactions with granule cells and climbing fibers. Generating Purkinje cells *in vitro* from iPSCs is especially challenging, given the complexity of cerebellar development and the lengthy maturation period required (Muguruma et al., 2015; Wang et al., 2024).

### Protocols for differentiating induced pluripotent stem cells into Purkinje cells

A general approach to induce cerebellar lineages from iPSCs emulates the posterior-hindbrain environment (Muguruma et al., 2015; Wang et al., 2015; Ishida et al., 2016; Sundberg et al., 2018; Watson et al., 2018; Buchholz et al., 2020): (1) Neural induction: SMAD inhibition generates neural precursors with broad anterior–posterior potential. (2) Posteriorisation: Moderate-to-high FGF signaling (FGF2, FGF8b) plus insulin promotes hindbrain identity (Wang et al., 2015; Ishida et al., 2016; Watson et al., 2018). Timed WNT modulation with CHIR-99021 (CHIR) further refines dorsal hindbrain patterning (Sundberg et al., 2018; Buchholz et al., 2020). (3) Maturation: Tri-iodothyronine (T3), NT-3, BDNF, and co-culture with granule cells, mouse cerebellar cells enhance survival and functional maturation.

Differentiation efficiency remains a significant challenge in the generation of Purkinje cells. These cells are typically identified by the expression of PCP2 (also known as L7) or calbindin, but without the use of purification techniques such as fluorescence-activated cell sorting or immunopanning, yields are generally low, ranging from 10% to 20%. The highest reported efficiency to date, 60%–90%, was achieved by Sundberg et al. (2018) using immunopanning to enrich the Purkinje cell population. Functionally, Purkinje cells are characterized by spontaneous high-frequency firing and complex spikes driven by climbing fiber input. While recreating these specific afferents *in vitro* remains technically challenging, patch-clamp studies have detected characteristic action potentials and synaptic responses when granule cell input is present (Wang et al., 2015).

Recently, increasing attention has turned to three-dimensional cerebellar organoid approaches to better replicate *in vivo*-like cytoarchitecture and cellular interactions (Muguruma et al., 2015; Muguruma, 2018; Silva et al., 2020; Atamian et al., 2024). Notably, Silva et al. (2020) and Atamian et al. (2024) succeeded in generating functionally mature Purkinje cells without the need for co-culture with mouse glial cells, granule cells, or cerebellar slices, marking a significant step forward in the development of fully human *in vitro* cerebellar models.

### Challenges and applications in disease modelling

Cerebellar disorders, notably spinocerebellar ataxias, are linked to Purkinje-cell dysfunction or loss. iPSC-derived Purkinje cells therefore offer a powerful platform for dissecting monogenic ataxias (Burman et al., 2021; Homma et al., 2024). However, reproducible generation of pure Purkinje populations, the protracted maturation timeline and the need for intricate synaptic connectivity remain major bottlenecks. Recent protocols report Purkinje-like neurons without granule-cell co-culture (Silva et al., 2020; Nayler et al., 2021; Madencioglu et al., 2022), yet efficiency and consistency vary. Advances in bioengineering—microfluidic platforms, bioreactors, and biomimetic scaffolds—and deeper insight into cerebellar-developmental cues are expected to overcome these limitations.

## Cholinergic Neurons

Cholinergic neurons are defined by their capacity to synthesize and release acetylcholine. They play critical roles in both central and peripheral nervous system functions, including memory, learning, arousal, motor control, and autonomic regulation. In the CNS, cholinergic neurons primarily reside in the basal forebrain, medial septum, and brain-stem nuclei. Peripherally, they comprise spinal motor neurons (see next section) and autonomic neurons such as parasympathetic ganglion cells. During embryogenesis, central cholinergic neurons arise from the ventral telencephalon and basal forebrain under the influence of SHH, FGFs, and BMP gradients. Key transcription factors include NKX2-1, LHX8, ISL1, and PHOX2B (Bissonnette et al., 2011; Crompton et al., 2013).

### Protocols for cholinergic-neuron induction

Differentiation of basal forebrain cholinergic neurons from iPSCs involves step-wise exposure to developmental morphogens (Hu et al., 2016b; Ortiz-Virumbrales et al., 2017; Muñoz et al., 2020; Wang et al., 2020; Krajka et al., 2021; Tao et al., 2024a).

A representative protocol comprises: (1) Neural induction & ventralization: dual-SMAD inhibition to establish neuroectoderm, followed by SHH agonists (SAG, purmorphamine, or recombinant SHH N-terminal fragment) to specify ventral forebrain/hindbrain identity. (2) Progenitor specification: co-application of FGF8 and BMP inhibitors drives cholinergic fate commitment. (3) Terminal differentiation: BDNF, GDNF, nerve growth factor (NGF), BMP9, IGF-1, CNTF, and dbcAMP support survival, neurite extension, and synaptogenesis.

The typical culture period required to generate functional cholinergic neurons is 50–60 days (**Additional Figure 1**, Cholinergic neuron). Cholinergic identity is commonly verified through immunostaining or gene expression analysis of key markers such as ChAT, VAChT, and acetylcholinesterase. In most published protocols, the purity of cholinergic neurons remains below 50% among TUBB3- or MAP2-positive cells. However, Muñoz et al. (2020) reported the highest induction efficiency to date, achieving 87% ChAT-positive cells within the TUBB3-positive population. Although overall purity remains a challenge in many studies, disease modeling efforts using cholinergic neurons have still proven valuable. Notably, Ortiz-Virumbrales et al. (2017), Wang et al. (2020), and Tao et al. (2024) reported Alzheimer’s disease-related phenotypes in cholinergic neurons derived from patient or genetically modified iPSC lines.

### Applications and challenges in disease modelling

iPSC-derived cholinergic neurons, especially basal forebrain cholinergic neurons, are crucial tools for modeling Alzheimer’s disease and studying neurodegenerative mechanisms. These neurons exhibit disease-relevant phenotypes, such as elevated amyloid beta 42/40 ratios, calcium signaling abnormalities, and reduced excitability—features observed in both familial and sporadic Alzheimer’s disease cases (Duan et al., 2014; Ortiz-Virumbrales et al., 2017; Moreno et al., 2018; Tao et al., 2024a). iPSC-basal forebrain cholinergic neurons have enabled the dissection of *PSEN2* mutation-related effects on neuronal physiology, and pharmacological studies showed insulin can rescue calcium flux and lower amyloid beta 42/40, highlighting therapeutic potential (Moreno et al., 2018). However, challenges include the long, multi-step differentiation protocols, variability in cholinergic marker expression (e.g., ChAT and VAChT), and limited reproducibility across iPSC lines (Duan et al., 2014; Ortiz-Virumbrales et al., 2017). Electrophysiological immaturity and inconsistent responses to stimuli or neurotoxins further complicate modeling efforts (Moreno et al., 2018). These issues underscore the need for improved protocols and functional validations to enhance the reliability of iPSC-derived cholinergic neurons in neurodegenerative research.

Despite progress, key hurdles remain: (1) Slow functional maturation and incomplete electrophysiological properties necessitate prolonged culture and optimized media; (2) Low purity due to heterogeneous differentiation, calling for improved transcription-factor programming and fluorescence-activated cell sorting/magnetic-activated cell sorting purification; (3) Scale-up constraints that hamper large-batch production for high-throughput screening or cell-therapy applications.

Continued refinement of patterning cues, transcription-factor cocktails, and bioprocessing strategies is expected to yield more efficient, scalable, and physiologically mature cholinergic-neuron cultures.

## Motor Neurons

Spinal motor neurons originate from the ventral domain of the developing spinal cord, where a finely tuned SHH gradient from the notochord/floor plate interacts with dorsal BMP/WNT signals to establish progenitor domains (Jessell, 2000). Within this ventral region, Olig2-positive progenitors exposed to intermediate SHH levels commit to the motor-neuron lineage and ultimately innervate skeletal muscle to control voluntary movement.

### Protocols for motor-neuron induction

Protocols for generating iPSC-derived motor neurons build on the seminal work of Wichterle et al. (2002) with numerous refinements (Dimos et al., 2008; Karumbayaram et al., 2009; Du et al., 2015; Shimojo et al., 2015; Hu et al., 2016a; Bianchi et al., 2018; Fujimori et al., 2018; Sances et al., 2018; Faye et al., 2020; Cutarelli et al., 2021): (1) Neural induction: dual-SMAD inhibition to generate neuroepithelial precursors. (2) Caudalization: retinoic acid posteriorizes NPCs toward spinal identity; high WNT agonist dosing can reinforce this fate. (3) Ventralization: SHH agonists (purmorphamine, SAG) at moderate concentrations specify the pMN domain; Olig2 marks motor-neuron progenitors. (4) Terminal differentiation: withdrawal of mitogens and addition of BDNF, GDNF, NT-3, and IGF-1 promote maturation. Notch inhibitors (DAPT and compound E) further enhance cell-cycle exit and neuronal yield (Du et al., 2015; Bianchi et al., 2018; Setsu et al., 2025).

Mature motor neurons express ISL1/2, HB9 (MNX1), and ChAT. Functionally, they form neuromuscular junctions when co-cultured with myotubes, and patch-clamp recordings reveal repetitive firing and cholinergic transmission (Ting et al., 2025).

To improve reproducibility and accelerate differentiation, many groups employ transcription-factor programming. The most common NIL cocktail (NGN2, ISL1, and LHX3) drives rapid motor-neuron specification, although alternatives using NGN2 alone or combinations such as ND1/ND2 have also been reported (Hester et al., 2011; Goparaju et al., 2017; Goto et al., 2017; Garone et al., 2019; Sepehrimanesh and Ding, 2020; Limone et al., 2023; Setsu et al., 2025). Most recently developed protocols achieve high purity, with over 80% of cells expressing motor neuron markers such as HB9 or ISL1 and exhibit functional properties within a relatively short timeframe, often within 3 to 4 weeks.

### Applications and challenges in disease modelling

iPSC-derived motor neurons are indispensable for modelling amyotrophic lateral sclerosis (ALS) and spinal muscular atrophy. Patient-specific lines harboring *superoxide dismutase 1* (*SOD1*) or *fused-in sarcoma* (*FUS*), *TAR DNA binding protein* (*TARDBP*, *TDP-43*), C9ORF72 mutations (ALS) or SMN1 deletions (spinal muscular atrophy) reproduce disease hallmarks such as axonal degeneration, protein aggregation, and RNA-processing defects (Ichiyanagi et al., 2016; Goto et al., 2017; Fujimori et al., 2018; Imaizumi et al., 2022; Ma et al., 2022; Morimoto et al., 2023; Setsu et al., 2025). These cultures serve as testbeds for small molecules, antisense oligonucleotides, and gene-editing strategies, offering translational insight. Although approximately 90% of ALS cases are sporadic, without a clear genetic inheritance pattern, relatively few iPSC-based disease modeling studies have focused on these cases. As motor neuron differentiation protocols continue to mature, achieving consistent induction periods and high purity, there is growing potential to apply iPSCs derived from sporadic ALS patients for disease modeling and drug screening. Expanding research in this area will be critical for capturing the broader pathological spectrum of ALS and developing more universally effective therapeutic strategies.

Persistent bottlenecks include limited long-term survival and heightened vulnerability to metabolic stress. Optimized trophic-factor cocktails, astrocyte or skeletal-muscle co-culture, and improved bioenergetic media have all extended viability and enhanced functional maturation, yet further work is needed to achieve fully adult-like, stress-resistant motor neurons *in vitro*.

## Dopaminergic Neurons

Dopaminergic (DA) neurons orchestrate motor control, motivation, and reward processing. Their dysfunction underlies several neurological and psychiatric disorders, most notably Parkinson’s disease, characterized by the progressive loss of mid-brain DA neurons in the substantia nigra pars compacta. Understanding how to generate DA neurons *in vitro* is therefore a major focus of stem-cell research for disease modelling, drug discovery, and cell-replacement therapy.

*In vivo*, mid-brain DA neurons arise from the ventral mesencephalon under the influence of SHH from the ventral mid-line and FGF8 from the mid–hind-brain boundary. These morphogens establish the floor-plate domain wherein transcription factors LMX1A, FOXA2, and NURR1 drive DA specification and maturation. Most differentiation protocols attempt to replicate these cues in a stepwise manner.

### Protocols for dopaminergic-neuron induction

A typical workflow begins with dual-SMAD inhibition to generate neuroectoderm, followed by a floor-plate patterning paradigm (Kriks et al., 2011; Kirkeby et al., 2012; Lin et al., 2016; Yamaguchi et al., 2020; Cardo et al., 2023): (1) Neural induction: dual-SMAD inhibition in chemically defined medium. (2) Ventral-mid-brain specification: SHH (or SAG/purmorphamine) plus FGF8 to induce FOXA2⁺/LMX1A⁺ floor-plate identity. (3) DA-progenitor expansion: maintenance in moderate SHH/FGF8 to stabilize FOXA2⁺/LMX1A⁺/OTX2⁺ progenitors. (4) Terminal differentiation: withdrawal of mitogens and addition of BDNF, GDNF, ascorbic acid, dbcAMP, and transforming growth factor-β3. NURR1 activity is pivotal for final DA maturation. Within 2–4 weeks, cells express tyrosine hydroxylase (TH) and dopamine transporter.

CHIR-mediated WNT activation further reinforces mid-brain patterning (Tao and Zhang, 2016).

An alternative strategy uses forced expression of LMX1A, FOXA2, NURR1, or PITX3 to accelerate conversion of NPCs into DA neurons (Ng et al., 2021; Nishimura et al., 2022; Sheta et al., 2022), although bypassing developmental checkpoints may compromise full physiological maturation. In contrast, Xue et al. (2019) adopted a different approach: their protocol employed transient expression of pan-neuronal transcription factors ATOH1 and NGN2, in combination with a cocktail of patterning factors including SHH, FGF8, and DAPT, to rapidly induce a dopaminergic neuron phenotype (Xue et al., 2019). This method aims to balance efficiency with developmental fidelity.

Dopaminergic identity is typically confirmed by immunocytochemistry for TH, while functional maturity is validated using electrophysiological assays such as patch-clamp recordings. Most published protocols report differentiation efficiencies of approximately 40%–60% TH-positive cells among the TUBB3-positive neuronal population. However, the most efficient protocol to date, reported by Xue et al. (2019), achieved over 90% TH-positive cells within the TUBB3-positive population, marking a significant advancement in dopaminergic neuron induction.

### Applications and challenges

iPSC-derived DA neurons have illuminated Parkinson’s disease pathogenesis, modelling patient-specific mutations such as LRRK2 and PARK2 (Parmar et al., 2020). These systems reveal mitochondrial deficits, α-synuclein aggregation, and autophagy impairment (Imaizumi et al., 2012; Reinhardt et al., 2013; Schöndorf et al., 2014; Ohta et al., 2015; Brazdis et al., 2020; Kouroupi et al., 2020; Bayati et al., 2024) and serve as platforms to screen neuroprotective compounds.

Key translational hurdles include: (1) Precise lineage specification and graft purity for cell-therapy applications; (2) long-term functional integration into host circuitry; (3) immune compatibility and manufacturing under GMP conditions; (4) ongoing ethical/regulatory considerations.

As differentiation protocols advance, there is growing emphasis on achieving functional maturation and subtype specificity, particularly in distinguishing A9 and A10 dopaminergic subpopulations. Within the midbrain, A9 dopaminergic neurons of the substantia nigra are critical for motor control and are selectively degenerated in Parkinson’s disease (Brichta and Greengard, 2014; Kamath et al., 2022). As such, A9 neurons represent a key target for both disease modeling and cell replacement therapies, underscoring the importance of refining protocols to generate subtype-specific dopaminergic neurons with high fidelity. To enhance the relevance and efficacy of iPSC-based applications, it is critical to establish more precise control over dopaminergic subtype differentiation and maturation (Moriarty et al., 2022).

Nevertheless, clinical translation is advancing. Pre-clinical and early-phase clinical trials show encouraging outcomes (Schweitzer et al., 2020; Kirkeby et al., 2023; Sawamoto et al., 2025; Tabar et al., 2025). BlueRock Therapeutics Inc. has initiated a Phase III trial of its iPSC-derived DA-neuron product, bemdaneprocel (BlueRock Therapeutics, 2025), marking a milestone for regenerative treatment of Parkinson’s disease.

## Serotonergic Neurons

Serotonergic neurons are concentrated in the dorsal and median raphe nuclei of the hindbrain. During embryogenesis, patterning relies on a delicate interplay of FGF, WNT, and retinoic-acid signals. The transcription factors FOXA2, LMX1B, and fifth Ewing variant (FEV) (PET1) are instrumental in specifying the serotonergic phenotype. FEV, for example, is crucial for the expression of genes required for serotonin synthesis and re-uptake, including tryptophan hydroxylase-2 (TPH2) and serotonin transporter (SERT).

### Protocols for serotonergic-neuron induction

A Recent approach to derive serotonergic neurons from iPSCs involves (Lu et al., 2016; Valiulahi et al., 2021): (1) Neural induction: dual-SMAD inhibition to generate neuroepithelial precursors. (2) Posterior-hindbrain patterning: FGF4 ± retinoic acid drives caudal identity; low-to-moderate WNT signaling supports posteriorization. (3) Ventralization: low-range SHH specifies ventral basal-plate territory. (4) Serotonergic maturation: BDNF, GDNF. and IGF-1, together with ascorbic acid and cAMP analogues, promote survival and serotonergic differentiation. Within 3–5 weeks, cells up-regulate.

A consistent method to validate iPSC-derived serotonergic neurons is immunostaining for serotonin itself in the cells, often alongside other neuronal markers (MAP2 and TUJ1) to ensure they are mature neurons.

The development of human-induced serotonergic neurons has progressed from transcription factor-driven conversion to developmentally guided, region-specific differentiation strategies. Early approaches employed forced expression of key genes such as *ASCL1*, *FOXA2*, *FEV*, *NKX2.2*, *GATA2*, and *LMX1B* to convert human fibroblasts or iPSCs into serotonergic neurons (Nefzger et al., 2011; Vadodaria et al., 2016; Xu et al., 2016b). Lu et al. (2016) developed a developmentally guided protocol that mimics embryonic hindbrain development using WNT, SHH, and FGF signaling pathways. This method successfully generated rostral hindbrain serotonergic neurons expressing canonical serotonergic markers, including TPH2, SERT, and VMAT2 (Lu et al., 2016; Jansch et al., 2021) (**Additional Figure 1**, Serotonergic neurons).

Recent advances have focused on subtype identity and functionality. Valiulahi et al. (2021) developed a protocol to generate caudal-type serotonergic neurons and hindbrain-like organoids using retinoic acid to pattern cells toward rhombomeres r5–r8 (Valiulahi et al., 2021). Although the differentiation efficiency of this approach was lower than that of Lu et al. (approximately 30% serotonin-positive/TUBB3-positive cells versus 60% in the Lu protocol), the resulting neurons exhibited robust serotonergic identity, fired action potentials, and released serotonin in response to pharmacological stimulation, including treatment with escitalopram.

### Applications and challenges

Given the pivotal role of serotonergic neurons in mood regulation, cognition, and numerous psychiatric disorders—including depression, anxiety, and obsessive-compulsive disorder—iPSC-derived serotonergic neurons provide a valuable *in vitro* platform for disease modelling and neuropsychiatric-drug screening (Licinio and Wong, 2016; Cao et al., 2017a; Jansch et al., 2021).

Key challenges remain: (1) Lack of quantifiable cellular read-outs for complex psychiatric phenotypes; (2) Heterogeneity and immature electrophysiological profiles, necessitating long-term culture or co-culture with astrocytes; (3) Scalability and purity constraints for high-throughput assays. (4) Addressing these limitations will require refined maturation protocols, multi-omic phenotyping, and development of functional assays that capture serotonergic network activity and neurotransmitter dynamics.

## Sensory Neurons

Sensory neurons of the peripheral nervous system originate from the neural crest and, to a lesser extent, from cranial placodes. The most widely studied iPSC-derived sensory neurons are dorsal-root-ganglion–like cells, which process nociceptive (pain), thermosensory, and mechanosensory stimuli. During embryogenesis, these neurons emerge at the neural-tube border, specified by transcription factors BRN3A, ISL1, and NGN-1/2.

### Protocols for sensory-neuron induction

Generating sensory neurons from iPSCs involves sequential neural-crest induction followed by sensory specification (Chambers et al., 2012; Young et al., 2014; Cai et al., 2017; Schwartzentruber et al., 2018; Deng et al., 2023): (1) Neural-crest induction: WNT activation (CHIR), SMAD inhibition (LDN, A83-01 or SB), and NOTCH blockade (dibenzazepine/DAPT) drive neuroepithelial cells toward a p75^NTR^/SOX10^+^ neural-crest state. (2) Sensory specification: exposure to NGF, BDNF, or NT-3 induces BRN3A^+^/ISL1^+^ dorsal-root-ganglion-like progenitors. (3) Maturation: prolonged culture with NGF, BDNF, and/or GDNF yields mature subtypes that express TRPV1, Nav1.8, and other modality-specific channels.

An alternative “direct-programming” route uses transient over-expression of NGN2 and/or BRN3A to accelerate differentiation (Nickolls et al., 2020; Plumbly et al., 2022).

The development of human iPSC-derived sensory neurons has advanced from early small-molecule-based protocols to more refined, scalable, and subtype-specific approaches. Most recent methods report over 70% differentiation efficiency based on BRN3A expression and demonstrate functional electrical activity (see **Additional Figures 1** and **2**, Sensory neuron). Initial methods used dual-SMAD inhibition and inhibitors such as CHIR, SU5402, and DAPT to generate nociceptor-like neurons expressing BRN3A, ISL1, and TrKA (Chambers et al., 2012; Cai et al., 2017; Schwartzentruber et al., 2018). These neurons responded to capsaicin and ATP, mimicking dorsal root ganglia nociceptors. However, outcomes were often variable between iPSC lines. Recently, Deng et al. (2023) further optimized protocols for high-throughput production, integrating robotic handling and quality control.

There is another strategy to improve reproducibility and subtype control. For example, inducible transcription factors such as NGN1/2 and BRN3A approaches were taken to drive differentiation into functionally distinct sensory neuron subtypes, including mechanoreceptors and nociceptors (Nickolls et al., 2020; Plumbly et al., 2022). These neurons displayed characteristic ion channel expression and functional excitability.

Large-scale efforts, such as Schwartzentruber et al., (2018), emphasized donor variability and culture conditions as major determinants of differentiation success. These protocols now support disease modeling, pain research, and drug screening, moving toward standardized, efficient production of human sensory neurons with defined phenotypes.

Collectively, these advancements are moving iPSC-derived sensory neurons toward greater reproducibility, subtype specificity, and utility in modeling human pain and sensory disorders.

### Applications and challenges

Inhibitory postsynaptic current-derived sensory neurons have emerged as a powerful tool in pain research, enabling human-relevant modelling of disorders such as inherited erythromelalgia, small fiber neuropathy, and hereditary sensory neuropathy type 1. By retaining patient-specific genetic backgrounds, induced sensory neurons allow researchers to replicate disease-associated neuronal hyperexcitability and evaluate drug responsiveness, including to Nav1.7 inhibitors and histamine antagonists (Clark et al., 2021; Labau et al., 2022; Hiranuma et al., 2024; Kalia et al., 2024). Electrophysiological and multielectrode array-based studies have linked specific ion channel mutations (e.g., Nav1.7, KCNQ2/3) to altered firing properties and pain phenotypes, underscoring their utility for genotype-phenotype analysis and personalized therapy (Meents et al., 2019; Mis et al., 2019; Yuan et al., 2021). Moreover, commercial platforms such as RealDRG™ enable high-throughput screening for analgesics, broadening their applicability to drug discovery (Kalia et al., 2024).

However, several challenges hinder widespread application. Differentiation protocols are time-consuming and prone to variability due to donor genetics and culture conditions (Cao et al., 2016; Lampert et al., 2020; Schweitzer et al., 2020; Labau et al., 2022). Induced sensory neurons often display immature phenotypes and reduced expression of key sensory markers such as TRPV1 or Nav1.8, limiting their fidelity to native dorsal root ganglion neurons (Labau et al., 2022; Hiranuma et al., 2024). Technical issues, including poor replating efficiency, space-clamp artifacts in electrophysiology, and inconsistent transcriptomic profiles, reduce reproducibility and scalability. Standardization, improved maturation methods, and rigorous validation are needed to fully harness the translational potential of induced sensory neurons in pain therapeutics.

## Noradrenergic (Norepinephrine) Neurons

Noradrenergic (NE) neurons in the central nervous system, primarily located in the locus coeruleus (LC) of the brainstem, modulate arousal, attention, and stress responses. Human LC noradrenergic neurons are of great interest for studying neurodegenerative diseases such as Parkinson’s and Alzheimer’s diseases (where LC degeneration is often observed) and psychiatric conditions such as anxiety and post-traumatic stress disorder (Weiss et al., 1994; Benarroch, 2009; Espay et al., 2014; Nobuta et al., 2015; Weinshenker, 2018). Until recently, the efficient generation of LC-like noradrenergic neurons from iPSCs remained an unmet challenge, owing to the complexity of the developmental cues required for this small yet diverse neuronal population (Robertson et al., 2013). However, recent breakthroughs have established differentiation protocols by building on prior work and systematically defining key conditions (Mong et al., 2014; Tao et al., 2024b).

### Protocols for noradrenergic-neuron induction

To date, only one study has reported efficient derivation of human LC-NE neurons (Tao et al., 2024b). Their three-step protocol achieves 40%–60 % NE neurons (22% TH^+^ NE neurons) from pluripotent stem cells: (1) Neural induction: dual-SMAD inhibition generates neuroepithelial cells; WNT activation with CHIR posteriorizes them toward the positional identity of hind-brain rhombomere1 (r1), where locus coeruleus neurons arise. (2) NE specification: ACTIVIN A is applied dose-dependently: 25 ng/mL up-regulates ASCL1, whereas 125 ng/mL induces PHOX2A/B, establishing NE progenitors. (3) Maturation: BDNF, GDNF, cAMP, and ascorbic acid support survival and functional differentiation.

Key transcription factors involved in the specification of locus coeruleus noradrenergic (LC-NE) neurons include ASCL1, PHOX2A, and PHOX2B. Single-nucleus RNA sequencing has confirmed a stepwise developmental trajectory from hindbrain progenitors to ASCL1-expressing precursors, culminating in PHOX2A- and PHOX2B-positive noradrenergic neurons. Functional assays further demonstrated activity-dependent norepinephrine release, indicating successful acquisition of neurotransmitter identity and physiological function.

### Applications and challenges

iPSC-derived LC-NE neurons provide models for neurodegenerative and psychiatric disorders (Alzheimer’s disease, Parkinson’s disease, attention deficit hyperactivity disorder, and depression). In a study by Tao et al. (2024b), a genetically encoded NE sensor (GRABNE1m) enables real-time monitoring and high-throughput drug screening. Additionally, transplantation into mouse brain showed axonal out-growth, underscoring regenerative potential. Remaining hurdles include raising yield and purity to clinical grade and ensuring long-term maturation and circuit integration after transplantation.

## Sympathetic Neurons

Sympathetic neurons, a branch of the autonomic nervous system, originate from neural-crest cells during embryogenesis. They regulate involuntary physiological responses such as heart rate, vasoconstriction, and gastrointestinal motility. Differentiation from human pluripotent stem cells therefore requires recapitulating neural-crest cues that instruct a sympathetic fate. Key transcription factors include PHOX2B, ASCL1, and HAND2, which drive specification and maturation.

### Protocols for sympathetic-neuron induction

As in sensory-neuron protocols, iPSC-derived sympathetic neurons are generated via a neural-crest intermediate (Oh et al., 2016; Zeltner et al., 2016; Frith et al., 2018; Kirino et al., 2018; Saito‐Diaz et al., 2019; Wu and Zeltner, 2020; Fan et al., 2024; Wu et al., 2024c): (1) Neural induction: dual-SMAD inhibition produces neuroepithelial precursors. (2) Neural-crest induction: WNT activation (CHIR) together with moderate, temporally controlled BMP signaling steers cells toward a p75^NTR^/SOX10^+^ neural-crest state. (3) Sympathetic specification & maturation: BMP2/4 exposure converts neural-crest cells into sympathetic progenitors; NGF, BDNF, GDNF, and cAMP analogues support survival, induce catecholaminergic enzymes, and boost norepinephrine synthesis.

Sympathetic neurons are identified by TH, dopamine-β-hydroxylase, and vesicular monoamine transporter 2 (VMAT2). Function is verified by stimulus-evoked norepinephrine release. To enrich sympathetic neurons, several studies, including those by Zeltner et al. (2016), Saito-Diaz et al. (2019), and Fan et al. (2024), have employed flow cytometry during the differentiation process. In contrast, the most efficient protocol based on TH expression was developed by Kirino et al. (2018), achieving up to 80% TH-positive cells without the need for any cell sorting procedures.

The development of induced sympathetic neurons from human PSCs has advanced significantly in recent years, transitioning from low-efficiency methods to highly reproducible and functionally validated protocols. Early differentiation attempts typically yielded mixed cell populations and relied on animal-derived serum and feeder layers, limiting scalability and reproducibility (Oh et al., 2016; Zeltner et al., 2016; Wu and Zeltner, 2019).

A critical advancement came with the recognition that trunk neural crest cells, derived via neuromesodermal progenitors, are the developmental origin of sympathetic neurons (Kirino et al., 2018; Frith and Tsakiridis, 2019). Efficient protocols now start by inducing neuromesodermal progenitors using WNT and FGF signaling, followed by BMP-mediated dorsalization to generate trunk neural crest cells, and then sympathetic lineage specification using BMP4, RA, and neurotrophic factors such as NGF, BDNF, and GDNF (Frith and Tsakiridis, 2019; Fan et al., 2024; Wu et al., 2024a).

Wu et al. (2024) and Fan et al. (2024) demonstrated robust generation of mature and functional sympathetic neurons using chemically defined, feeder-free conditions. These sympathetic neurons express key markers (TH, dopamine-β-hydroxylase, PHOX2B), exhibit electrophysiological activity, and secrete norepinephrine.

### Applications and challenges

Induced sympathetic neurons derived from human iPSCs offer significant promise for studying human autonomic biology, cardiovascular modulation, and neurocardiac disease modeling. These cells can form functional connections with cardiomyocytes, enabling *in vitro* modeling of sympathetic modulation of heart rate. Notably, cocultures of human iPSC-derived sympathetic neurons and cardiomyocytes have demonstrated that norepinephrine secretion from neurons can increase cardiac beating rates upon nicotine stimulation, mimicking neurocardiac junction behavior (Winbo et al., 2020; Bernardin et al., 2022). Additionally, such systems have shown utility in assessing electrophysiological properties, neuronal maturation, and the impact of sympathetic input on cardiac structure and function (Oh et al., 2016). Moreover, these platforms are suitable for drug screening and disease modeling, including for inherited arrhythmia syndromes and autonomic dysfunction (Saito‐Diaz et al., 2019).

However, several challenges remain. Differentiation efficiency is variable, often requiring reporter systems or fluorescence-activated cell sorting purification to enrich sympathetic lineages (Zeltner et al., 2016; Saito‐Diaz et al., 2019). Protocols tend to be complex and lengthy, and the resultant neurons often display immature phenotypes, lacking the full electrophysiological or molecular characteristics of adult human neurons (Wu and Zeltner, 2019). Coculture with target tissues such as cardiomyocytes enhances maturation, but such systems are technically demanding and not readily scalable (Wu and Zeltner, 2019; Winbo et al., 2020; Bernardin et al., 2022). Additionally, standardization across labs remains a hurdle, especially with iPSC lines from diverse genetic backgrounds. Achieving postnatal maturity and functional integration with target tissues *in vitro* remains a key future goal.

## Parasympathetic Neurons

Parasympathetic neurons are a core component of the autonomic nervous system, mediating “rest-and-digest” responses via acetylcholine release. Developmentally, they arise from Schwann-cell precursors—a lineage distinct from the neural-crest–derived pathway that produces sympathetic neurons (Dyachuk et al., 2014; Espinosa-Medina et al., 2014). This unique origin underpins their cranial and sacral innervation patterns and their roles in gland secretion, cardiovascular regulation and organogenesis.

### Protocols for parasympathetic-neuron induction

Wu et al. (2024) established a developmentally faithful protocol: (1) Neural-crest induction: human pluripotent stem cells are directed to SOX10^+^ neural-crest cells. (2) Schwann-cell precursor formation: cells are aggregated as spheroids in CHIR, FGF2, and NRG1. (3) Parasympathetic neuronal specification: replating Schwann-cell precursors in medium containing BDNF, GDNF, CNTF, retinoic acid, dbcAMP, and low serum; CNTF is indispensable for parasympathetic neuronal fate.

An alternative method by Takayama et al. (2020) co-induces sympathetic and parasympathetic neurons from iPSC-derived neural-crest cells. High cell density biases toward sympathetic fate, whereas elevated BDNF and CNTF levels favor parasympathetic neurons. Co-culture with iPSC-derived cardiomyocytes showed reciprocal regulation of beating rate, underscoring the platform’s utility for modelling cardiac autonomic control.

Parasympathetic neurons are identified by ChAT, VAChT, neuronal nitric oxide synthase, PHOX2B, peripherin, and the parasympathetic-specific transcription factor HMX3, with minimal glial contamination.

### Applications and challenges

Human pluripotent stem cell-derived parasympathetic neurons enable human-specific modeling of autonomic function and disease (Takayama et al., 2023; Akagi et al., 2024). Recent protocols, such as that by Wu et al. (2024), generate functional parasympathetic neurons via Schwann cell precursors, exhibiting cholinergic identity, spontaneous activity, and responses to muscarinic agonists. These models have been used to study familial dysautonomia, Sjögren’s syndrome, and coronavirus disease 2019-related autonomic dysfunction, and show utility in neuro-cardiac and adipose co-cultures (Wu et al., 2024b). However, challenges persist. Differentiation is complex and time-consuming, often yielding heterogeneous populations. Functional maturity and subtype specificity remain limited, and standard markers are insufficiently defined. As reviewed in Bos et al. (2025), few protocols achieve high purity or demonstrate consistent electrophysiological validation (Bos et al., 2025). Refinement of signaling cues and functional assays is essential for advancing translational applications.

## Conclusions and Future Directions

Since 2015, advances in iPSC differentiation protocols have enabled the generation of an impressive spectrum of human neuronal subtypes *in vitro*, faithfully reflecting many major classes of neurons found in the nervous system. Each subtype, glutamatergic, GABAergic, dopaminergic, serotonergic, motor, sensory, sympathetic, parasympathetic, Purkinje, cholinergic, and noradrenergic, requires a tailored combination of developmental signals or transcriptional cues, and researchers have developed innovative strategies to meet these needs. The diversity of approaches highlights that no single method is universally applicable; rather, protocols are specialized to recapitulate the embryonic origin of each neuron type. Encouragingly, many methods are now converging toward being reproducible, efficient, and even scalable. Rapid differentiation protocols, producing glutamatergic neurons, dopaminergic neurons, and motor neurons within days to weeks, are particularly advantageous for high-throughput studies and time-sensitive experiments. In contrast, more extended protocols, such as those for Purkinje cells or basal forebrain interneurons, yield highly specialized neurons but require weeks to months of culture, necessitating careful consideration of their benefits in maturity and specificity relative to time and resource investment.

A recurring theme in these advancements is the stepwise implementation of developmental patterning, often substituting protein growth factors with small molecules to improve efficiency and consistency. Many protocols now employ all-chemical induction (e.g., dual-SMAD inhibition, GSK3β inhibitors, SHH agonists, retinoids), enhancing standardization and suitability for clinical-grade manufacturing. Another major trend is the use of transcription factor overexpression to accelerate neuronal differentiation. While this approach expedites neuron generation, it may bypass intermediate progenitor stages critical for proper integration or subtype diversity. Recent strategies increasingly balance speed and authenticity by combining transient expression of fate-determining factors (e.g., NGN2 for generic neurons, ASCL1/PET1 for serotonergic neurons) with developmental cues.

Consistency in characterizing differentiated neurons has also markedly improved. Almost all studies now rely on immunofluorescence and gene expression analyses of key marker panels specific to each neuronal subtype, with many incorporating electrophysiological validation to confirm that derived neurons are electrically active and form synapses. The universal adoption of such assays, particularly immunostaining for definitive markers, provides confidence that iPSC-derived cells genuinely represent their intended neuron types. This convergence in validation standards is a critical advancement for the field.

Despite this remarkable progress, challenges remain. Differentiation efficiency and purity vary among protocols and cell lines; even “high-yield” methods often produce cultures containing 40%–50% off-target cells or progenitors. Line-to-line variability in differentiation propensity is another area of active investigation, as certain genetic backgrounds or reprogramming methods may predispose iPSCs to specific fates. Moreover, many iPSC-derived neurons correspond to prenatal or early postnatal stages, raising concerns about their suitability for modeling adult-onset diseases.

To address these ongoing challenges, efforts are increasingly focused on the standardization of protocols, automation of culture systems, rigorous quality control, and the promotion of functional maturation in iPSC-derived cells. One of such approaches are automated culture platforms and robotic handling systems. Automation reduces manual error, enhance scalability, and support high-throughput differentiation—particularly important for clinical and drug screening applications.

Equally critical is the development of robust quality-control frameworks, which include early screening of iPSC lines for genetic and epigenetic stability, as well as real-time monitoring of culture conditions using biosensors for pH, oxygen, and metabolic activity. These tools help maintain consistency and detect protocol deviations early.

Finally, promoting the functional maturation of iPSC-derived cells remains an active frontier. Advanced culture platforms, including three-dimensional organoids, organ-on-chip systems, and dynamic bioreactors, are being used to better mimic native developmental environments. Electrical stimulation, co-culture with astrocytes or endothelial cells, and epigenetic reprogramming modulation by small molecules have also been shown to enhance electrophysiological and metabolic maturity (Oh et al., 2021; Hergenreder et al., 2024). Together, these innovations are laying the foundation for more robust, scalable, and clinically relevant iPSC-derived models.

In the context of major neurodegenerative diseases, cell-based therapeutic approaches diverge significantly, reflecting disease-specific pathological mechanisms. In Parkinson’s disease, the selective loss of dopaminergic neurons in the substantia nigra leads to dopamine deficiency and motor dysfunction. Accordingly, transplantation of dopaminergic progenitors derived from human embryonic stem cells or iPSCs has emerged as a promising strategy to restore dopaminergic tone and motor function (Kirkeby et al., 2025). In contrast, ALS and Alzheimer’s disease involve widespread neuronal degeneration, precluding simple cell replacement. In ALS, although lower motor neuron loss is central, functional recovery is limited by the inability of transplanted neurons to regenerate long-distance axons. Thus, current strategies focus on transplanting neural progenitor cells engineered to secrete neurotrophic factors (e.g., GDNF) to exert paracrine neuroprotection (Baloh et al., 2022). Similarly, in Alzheimer’s disease, extensive neuronal loss and amyloid and tau pathology complicate replacement efforts, shifting therapeutic approaches toward modulating neuroinflammation and providing trophic support via transplantation of neural stem cells or mesenchymal stem cells (Ou et al., 2024). Thus, while Parkinson’s disease represents a rare case where targeted cell replacement is feasible, therapeutic strategies for ALS and Alzheimer’s disease focus predominantly on neuroprotection and disease environment modulation. For translational research utilizing iPSC-derived neurons, it is crucial to align differentiation strategies with disease-specific requirements to maximize clinical impact.

As the primary focus of this review is the directed differentiation of specific neuronal subtypes, the application of iPSC-derived NPCs is not covered in detail here and has been comprehensively reviewed elsewhere (Sugai et al., 2025). Nonetheless, NPC transplantation represents another promising avenue for iPSC-based therapies, particularly in the context of spinal cord injury and traumatic brain injury. Preclinical studies have demonstrated encouraging results (Nori et al., 2011; Kobayashi et al., 2012), and clinical trials in humans are currently underway (Sugai et al., 2021).

In conclusion, the period since 2015 has witnessed transformative improvements in the derivation of specialized neuronal subtypes from human iPSCs. Researchers now have a comprehensive toolbox of protocols to generate neuronal types suited to diverse applications, from modeling neurodevelopmental disorders with cortical neurons to studying Parkinson’s disease with dopaminergic neurons or neuropathies with peripheral neurons. These advances bring the field closer to realizing the full potential of iPSC-derived neurons for regenerative medicine and drug discovery. Going forward, continued refinement and comparative head-to-head evaluations of protocols will be invaluable for establishing best practices for each neuronal subtype. Additionally, constructing more complex neural circuits by combining multiple iPSC-derived subtypes represents an exciting frontier for achieving greater physiological relevance *in vitro*. The achievements summarized here underscore a promising future for neural regeneration research and disease modeling, powered by ever-advancing methodologies for iPSC-based neuronal differentiation.

## Additional files:

***Additional Figure 1:*** *Induction methods based on patterning and developmental cues.*

Additional Figure 1 Induction methods based on patterning and developmental cues.
This schematic summarizes key protocols for differentiating iPSCs into neuronal subtypes using region-specific patterning strategies that mimic embryonic developmental cues. The timeline of induction, reported efficiencies, functional assay methods, and total differentiation duration are illustrated for each protocol. Illustration created by LAIMAN Inc. and used with permission under purchased license. AA: Ascorbic acid; BDNF: brain-derived neurotrophic factor; bFGF: basic fibroblast growth factor; BMP2: bone morphogenetic protein 2; BMP4: bone morphogenetic protein 4; BMP9: bone morphogenetic protein 9; BSA: bovine serum albumin; cAMP: cyclic adenosine monophosphate; CHIR: CHIR-99021; CNTF: ciliary neurotrophic factor; dbcAMP: dibutyryl-cAMP; DBZ: dibenzazepine; DM: dorsomorphin; dox: doxycycline; EGF: epidermal growth factor; FGF2: fibroblast growth factor 2; FGF4: fibroblast growth factor 4; FGF8b: fibroblast growth factor 8b; GDNF: glial cell line-derived neurotrophic factor; hLIF: human leukemia inhibitory factor; IGF-1: insulin-like growth factor 1; LDN: LDN-193189; NGF: nerve growth factor; NRG-1: neuregulin 1; NT-3: neurotrophin 3; PD: PD173074; RA: retinoic acid; RO: RO4929097; SAG: smoothened agonist; SB: SB431542; SHH: sonic hedgehog; SU: SU5402; T3: tri-iodothyronine; VPA: valproic acid; XAV: XAV-939.

***Additional Figure 2:*** *Transcription factors overexpression-based induction methods.*

Additional Figure 2 Transcription factors overexpression-based induction methods.
This diagram presents representative strategies for neuronal induction through direct transcription factor overexpression. Induction timelines, efficiencies, functional validation approaches, and protocol durations are indicated for selected neuronal subtypes. Illustration created by LAIMAN Inc. and used with permission under purchased license. For abbreviations, see legend to Additional Figure 1.

## Data Availability

*All relevant data are within the paper and its Additional files.*
